# Phytoestrogen *β*-Sitosterol Exhibits Potent In Vitro Antiviral Activity against Influenza A Viruses

**DOI:** 10.3390/vaccines11020228

**Published:** 2023-01-19

**Authors:** Sara Shokry, Akram Hegazy, Ahmad M. Abbas, Islam Mostafa, Ibrahim H. Eissa, Ahmed M. Metwaly, Galal Yahya, Assem M. El-Shazly, Khaled M. Aboshanab, Ahmed Mostafa

**Affiliations:** 1 Center of Scientific Excellence for Influenza Viruses, National Research Centre, Giza 12622, Egypt; 2 Department of Microbiology and Immunology, Faculty of Pharmacy, Ain Shams University, Abbassia, Cairo 11566, Egypt; 3 Department of Agricultural Microbiology, Faculty of Agriculture, Cairo University, Giza District, Giza 12613, Egypt; 4 Department of Microbiology and Immunology, Faculty of Pharmacy, King Salman International University (KSIU), Sinai 46612, Egypt; 5 Department of Pharmacognosy, Faculty of Pharmacy, Zagazig University, Zagazig 44519, Egypt; 6 Pharmaceutical Medicinal Chemistry & Drug Design Department, Faculty of Pharmacy (Boys), Al-Azhar University, Cairo 11884, Egypt; 7 Pharmacognosy and Medicinal Plants Department, Faculty of Pharmacy (Boys), Al-Azhar University, Cairo 11884, Egypt; 8 Biopharmaceutical Products Research Department, Genetic Engineering and Biotechnology Research Institute, City of Scientific Research and Technological Applications (SRTA-City), Alexandria 21934, Egypt; 9 Department of Microbiology and Immunology, Faculty of Pharmacy, Zagazig University, Zagazig 44519, Egypt; 10 Faculty of Pharmacy, El Saleheya El Gadida University, El Saleheya El Gadida 44813, Sharkia, Egypt

**Keywords:** respiratory viruses, COVID-19, influenza, *β*-sitosterol, molecular docking (MD)

## Abstract

Influenza is a contagious infection in humans that is caused frequently by low pathogenic seasonal influenza viruses and occasionally by pathogenic avian influenza viruses (AIV) of H5, H7, and H9 subtypes. Recently, the clinical sector in poultry and humans has been confronted with many challenges, including the limited number of antiviral drugs and the rapid evolution of drug-resistant variants. Herein, the anti-influenza activities of various plant-derived phytochemicals were investigated against highly pathogenic avian influenza A/H5N1 virus (HPAIV H5N1) and seasonal low pathogenic human influenza A/H1N1 virus (LPHIV H1N1). Out of the 22 tested phytochemicals, the steroid compounds *β*-sitosterol and *β*-sitosterol-*O*-glucoside have very potent activity against the predefined influenza A viruses (IAV). Both steroids could induce such activity by affecting multiple stages during IAV replication cycles, including viral adsorption and replication with a major and significant impact on the virus directly in a cell-free status “viricidal effect”. On a molecular level, several molecular docking studies suggested that *β*-sitosterol and *β*-sitosterol-*O*-glucoside exhibited viricidal effects through blocking active binding sites of the hemagglutinin surface protein, as well as showing inhibitory effects against replication through the binding with influenza neuraminidase activity and blocking the active sites of the M2 proton channel activity. The phytoestrogen *β*-sitosterol has structural similarity with the active form of the female sex hormone estradiol, and this similarity is likely one of the molecular determinants that enables the phytoestrogen *β*-sitosterol and its derivative to control IAV infection in vitro. This promising anti-influenza activity of *β*-sitosterol and its *O*-glycoside derivative, according to both in vitro and cheminformatics studies, recommend both phytochemicals for further studies going through preclinical and clinical phases as efficient anti-influenza drug candidates.

## 1. Introduction

Annual epidemics resulting from viral respiratory infections such as the common cold and influenza-like sicknesses lead to tragic impacts on global public health. They contribute substantially to high rates of morbidity and mortality worldwide [1]. The current COVID-19 pandemic serves as an example of how RNA viruses generate human, animal, and zoonotic infections that afflict millions of people. Viral respiratory infections are the utmost reason to seek health care in developing and developed countries [2,3]. Infections caused by respiratory viruses kill about 5 million children (<5 years) every year all over the world [4].

There is a huge number of respiratory viruses (>200 viruses) belonging to six families, namely *Adenoviridae*, *Herpesviridae, Picornaviridae, Orthomyxoviridae, Paramyxoviridae*, and *Coronaviridae* [5]. Nonetheless, family members of *Orthomyxoviridae* (especially influenza viruses) and *Coronaviridae* (especially severe acute respiratory syndrome coronavirus (SARS-CoV), Middle East respiratory syndrome (MERS), and SARS-CoV-2) have attracted more attention in the past two decades [6,7]. Seasonal outbreaks, endemic infections, and suddenly occurring pandemic situations are felt mainly within these two families [8,9,10]. All ages are susceptible to infection with influenza viruses and coronaviruses; however, young children (<5 years) and aged people (>65 years) have the highest incidence rate and may suffer more [11,12].

On a global scale, the reported number of severe cases caused only by influenza epidemics is about 3 to 5 million people, causing about 291,243 to 645,832 fatal cases. In the United States, cost-of-illness (COI) studies revealed an annual economic loss of about USD 87.1 billion [13,14]. Every 5 to 10 years, a new influenza pandemic event catches everyone off guard. It is mainly driven by the emergence of novel flu strains belonging to genus A of influenza viruses (IAV) [15]. Both influenza viruses and coronaviruses share the common features of being enveloped viruses with single-stranded RNA genomes. Unlike DNA viruses, RNA viruses are more prone to evolution, as they do not possess replication machinery with proofreading ability, which introduces continual changes to the viral genome, resulting in new strains [5,16].

A lack of immunity in individuals to a new viral strain(s) complicates infection control, ending with a global pandemic [17]. The postexposure use of antiviral drugs to cure respiratory viral infections is one of the successful chemoprophylaxis approaches [18]. Biologically active plant-metabolized products and plant extracts (e.g., flavonoids, terpenoids, steroids, polyphenols, saponins, coumarins, and lignans) are considered effective and safe therapeutic tools to combat respiratory viral infections [19,20,21,22]. The pharmacological pipeline for the prevention and treatment of prospective flu-like outbreaks will be widened by further investigation of these phytochemical medicines for clinical use [21]. In this study, different phytochemical classes were screened for their antiviral potential against avian and seasonal IAVs of subtypes H1N1 and H5N1 to find out safe, novel, and potent anti-influenza drug candidates. In addition, the stages of antiviral action of certain plant steroids (*β*-sitosterol and *β*-sitosterol-*O*-glucoside) that show promising anti-influenza activity have been investigated against the seasonal influenza A/H1N1 virus.

## 2. Materials and Methods

### 2.1. Cell Lines and Viruses

Madin Darby Canine Kidney (MDCK) cell line was obtained from the cell culture collection of the Center of Scientific Excellence for Influenza Viruses (CSEIV), National Research Centre (NRC), Cairo, Egypt, and were grown into monolayer culture using Dulbecco’s modified Eagle’s medium (DMEM) (DMEM; BioWhittaker, Walkersville, MD, USA) supplemented with 1% Penicillin/Streptomycin (pen/strep) antibiotic/antimycotic mixture (GIBCO-BRL; New York, NY, USA) and 5% fetal bovine serum (FBS) (Gibco-BRL; New York, USA). The cells were serially passaged and plated into 96-well, 12-well, and 6-well growth plates for cytotoxicity, plaque reduction, and mode of action, and proliferated at 37 °C in a humified environment with 5% CO_2_ whenever the monolayers were confluent. Consistently, two strains of IAVs, namely the highly pathogenic avian influenza A/chicken/Egypt/N12640A/2016 (H5N1) and seasonal influenza A/Egypt/NRC098/2019(H1N1) (GISAID ID: EPI_ISL_12995118) provided by the CSEIV, NRC, Egypt, were routinely grown in MDCK cells and/or specific pathogen-free (SPF) embryonated chicken eggs (ECE) [23,24]. Supernatants of infected cells were used to create virus stock cultures, which were then stored at −80 °C for short-term use. The viruses were titrated using median tissue culture infectious dose (TCID50) assay and plaque infectivity assay (PIA) as previously described [20].

### 2.2. Phytochemicals

Silybin, 7-hydroxyflavone, flavanone, saponin, lupeol, gluconic acid, galacturonic acid, D-sorbitol, digitonin, arbutin, D- (-) salicin, kaempferitrin, isoquercitrin, chrysophanic acid, aloe-emodin, o-coumaric acid, and vanillin were purchased from Sigma, St. Louis, MO, USA. Pinocembrin, *β*-sitosterol, and *β*-sitosterol-*O*-glucoside were isolated from *Centaurea eryngioides* [25]. Glucuronic acid and ouabain were obtained from Serva, Feinbiochemica, Heidelberg, Germany. Naringin was isolated from the peel of *Citrus jambhiri* Lush. fruit [26]. The investigated phytochemicals in the current study are discussed in Table 1.

### 2.3. Cytotoxicity and Antiviral Assay

Crystal violet assay, described earlier [102,103], was employed to determine the cytotoxic range of concentrations for each the tested compounds on the predefined cell lines through CC_50_ determination and to primarily investigate their antiviral potential against the IAVs and SARS CoV-2. Initially, 96-well cell culture plates were seeded with MDCK cells at cell density of 1 × 10^5^ cells/mL and incubated overnight under humified conditions at 37 °C in 5% CO_2_ atmosphere. Then, the cells were washed with 1x sterile DPBS and the compounds under investigation were serially added to the plates in tenfold dilution with triplicates while cell control wells were included. The plates were incubated in humified incubator at 37 °C with a 5% CO_2_ atmosphere for 3 days. Following incubation period, the plates underwent cell fixation with paraformaldehyde (10%) and visualization of CPE was then employed using the crystal violet stain (0.1%). Following routine washing with water, as previously mentioned, the plates were then left to dry overnight at 25 °C (room temperature). A volume of 100 µL of methanol (99.85%) was added over the stained cells to dissolve the crystal violet stain and to produce an optical density (OD), which was then measured using Anthos Zenyth 200rt reader (Anthos Labtec Instruments, Heerhugowaard, Netherlands) at a wavelength of 570 nm. In the same context, to assess the antiviral potential of the tested compounds, IC_50_ values were determined for each compound. Likewise, the cell lines (MDCK and Vero cells) were propagated in 96-well cell culture plates with the same densities as mentioned before. Viral adsorption step was conducted for 1 h at RT after routinely washing the cultured cells with sterile 1x DPBS. Immediately, 100 μL/well of each safe concentration (non-cytotoxic) for each compound was added to the cells, where cell and virus control wells were included, then the plates underwent a longer incubation time at 37 °C with 5% CO_2_ conditions in a humified incubator for 3 days. The plates then went through the same procedures of fixation, visualization, and OD measurement as in CC_50_ determination protocol.

### 2.4. Plaque Reduction Assay (PRA)

To ascertain the antiviral potential of the highly promising steroid compounds, the plaque reduction assay [103] was carried out with minor changes. In brief, viral dilutions were added to a range of nontoxic concentrations for each compound and incubated at RT for 1h. The mixture was then added in triplicates to confluent monolayers of MDCK cells (80–90% confluency), previously proliferated 12-well cell culture plates for 24 h, and the plates were then incubated at 37 °C with CO_2_ atmosphere to allow for viral adsorption onto host cell receptors. In the meantime, the plates were manually shaken smoothly at 15 min interval. Aspiration of residual inocula and washing with 1x sterile DPBS were then employed. Moreover, the plates were overlaid with 1% agarose and 1× DMEM as 2× overlay medium supplemented with 4 % BSA, 1% pen/strep mixture, and 1 µg/mL TPCK-treated trypsin (in case of working with H1N1 virus) and allowed to set. A longer incubation period for 60–72 h was carried out in humified incubator at 37 °C with 5% CO_2_ atmosphere. The same procedures of cell fixation and plaque visualization were handled as in plaque infectivity assay. The viral reduction percentage for each compound was then calculated according to the following equation [103]:

$$\mathrm{Viral}\;\mathrm{reduction}\;\left(\%\right)=\frac{\mathrm{Count}\;\mathrm{of}\;\mathrm{untreated}\;\mathrm{virus}\;\left(\mathrm{control}\right)-\;\mathrm{Count}\;\mathrm{of}\;\mathrm{treated}\;\mathrm{virus}}{\mathrm{Count}\;\mathrm{of}\;\mathrm{untreated}\;\mathrm{virus}\;\left(\mathrm{control}\right)}\;\times \;100$$

### 2.5. Stage(s) of the Antiviral Action

The stages at which the steroids (*β*-sitosterol and *β*-sitosterol-*O*-glucoside) with promising anti-influenza activity have been investigated against the seasonal influenza A/H1N1 virus. The three investigated targets for the antiviral action(s) are (1) the viral adsorption onto the host-cell receptor preventing virus adhesion, (2) the viral replication inside the host cells, and (3) the targeting of the viral particles away from the cell (cell-free viricidal effect). The impact of the potent compound on each of the three predefined stages was investigated using modified protocols of plaque reduction assays as described previously [20].

### 2.6. Data Collection and Heatmap Construction

The data covering sex-disaggregated numbers of influenza deaths during 2015, 2018, and 2019 were retrieved from the EU’s standardized death rate for diseases of respiratory system source Eurostat [104]. The heatmaps were created using the Clustvis online tool [105]; countries with constant numbers of both sexes were removed from the heatmap during the data processing.

### 2.7. In Silico Docking Studies

#### 2.7.1. Protein Preparation

The crystal structures of influenza hemagglutinin H1 mutant DH1E (PDB ID: 5VMG, resolution: 2.45 Å), neuraminidases (PDB ID: 3TI5, resolution: 1.90 Å), proton channel M2 protein (PDB ID: 2RLF), and hemagglutinin head epitope of influenza H1N1 virus (PDB ID: 7MEM) were obtained from Protein Data Bank (https://www.rcsb.org) (accessed on 10 December 2022). At first, the crystal structures of the selected proteins were prepared by removing crystallographic water molecules. Only one chain for each protein was retained besides the cocrystallized ligands. For influenza M2 proton channel protein, we used all chains in the docking process. Protein chains were protonated using the following setting. The used electrostatic functional form was GB/VI with a distance cut-off of 15 Å. The used value of the dielectric constant was 2 with an 80 dielectric constant of the used solvent. The used Van der Waals functional form was 800R3 with a distance cut-off of 10 Å. Then, minimization of energy was carried out. Next, the active pockets of different proteins were determined. The residues in the proteins that were within 5 Å of the cocrystallized ligand’s edge were identified as the active sites [106].

#### 2.7.2. Ligand Preparation

Structures of the tested compounds were drawn using ChemBioDraw Ultra 14.0 and saved in MDL-SD file format. These were protonated and optimized by energy minimization using MM2 force field [107].

#### 2.7.3. Docking Setup and Validation of Docking Protocol

MOE version 2019 was used in the docking studies. To validate the docking procedure, redocking of the cocrystallized ligands was carried out against the different active sites. Then, the produced RMSD values were calculated. The value less than 2 Å indicates the validity of the docking processes [108].

The docking procedures were carried out against the active sites producing setup for the 30 docked poses for each ligand using ASE as a scoring function [109]. The pose with good binding mode was selected. Discovery Studio (DS) 4.0 was used for visualization step [110]. Comparing the binding mode of the tested compounds with that of the reference molecules gives good insight about the binding pattern of the tested compounds [111,112].

### 2.8. Statistical Analysis

Using GraphPad Prism software version 5.04 (GraphPad Software Inc., La Jolla, CA, USA), statistical analysis was conducted using two-tailed unpaired T-tests. A value of *p* ≤ 0.05 was considered to indicate statistical significance.

## 3. Results

### 3.1. Cytotoxicity and Antiviral Potential of the Investigated Compounds

**Figure 1:**
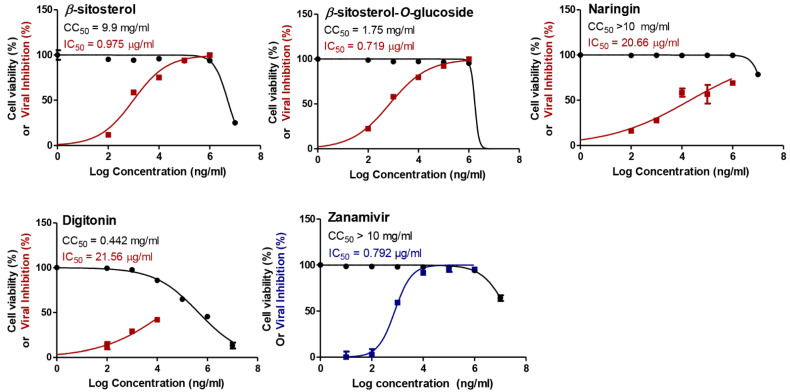
The cytotoxicity as expressed in CC_50_ (half-maximal cytotoxic concentration) and the antiviral efficacy against A/H1N1 as expressed in IC_50_ (half-maximal inhibitory concentration) for the studied phytochemicals. GraphPad Prism 5.01 software was used to analyze the nonlinear regression while the CC_50_ and IC_50_ were determined by plotting log inhibitor against normalized response (variable slope).

Different classes of phytochemical compounds were chosen based on their reported biological activities (Table 1), and a preliminary screening was carried out in order to evaluate their possible antiviral efficacy against the IAVs after assessing their toxic effects on the MDCK cells. The anti-influenza activities of the tested compounds were compared to the reference zanamivir drug that represents the main antiviral class against influenza, namely neuraminidase inhibitors (NAIs). Notably, almost all tested compounds showed reliable safe use on the tested cell line, with concentrations reaching up to 10 mg/mL for most of the compounds (Figure 1 and Supplementary Figure S1). The antiviral potential of these compounds was first evaluated against the seasonal human influenza A/H1N1 virus and compared with the predefined drug control.

Strikingly, the steroid compounds *β*-sitosterol and *β*-sitosterol-*O*-glucoside clearly exerted highly promising antiviral activities against the tested A/H1N1 virus, with IC_50_ values of 0.975 and 0.719 µg/mL, respectively (Figure 1).

On the other hand, naringin, kaempferitrin, lupeol, and digitonin (Figure 1) moderately showed their antiviral potential against the tested IAV, with IC_50_ values equal to 20.66, 47.8, 93.68, and 21.56 µg/mL, respectively. Unfortunately, the rest of the compounds elucidated poor or no antiviral activities against the HPAIV (A/H5N1) when compared to the drug control (Figure S1). According to the SI values (Table 2), the highly efficacious steroids (*β*-sitosterol and *β*-sitosterol-*O*-glucoside) were assessed for their anti-influenza potential against the highly pathogenic avian influenza A/H5N1 virus, so as to depict their broad-spectrum use against different IAV subtypes.

**Figure 2:**
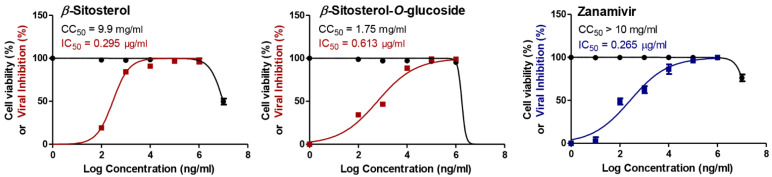
The cytotoxicity as expressed in CC_50_ (half-maximal cytotoxic concentration) and the antiviral potential against A/H5N1 as expressed in IC_50_ (half-maximal inhibitory concentration) for the most efficacious steroids (*β*-sitosterol and *β*-sitosterol-*O*-glucoside). GraphPad Prism 5.01 software was used to analyze the nonlinear regression, while the CC_50_ and IC_50_ were determined by plotting log inhibitor against normalized response (variable slope).

Remarkably, *β*-sitosterol and *β*-sitosterol-*O*-glucoside exhibited similar activities against the A/H5N1 virus with lower IC_50_ values of 0.295 and 0.613 µg/mL, respectively, when compared to the zanamivir as drug control (Figure 2).

### 3.2. Viral titer Reduction in a Concentration-Dependent Manner

**Figure 3:**
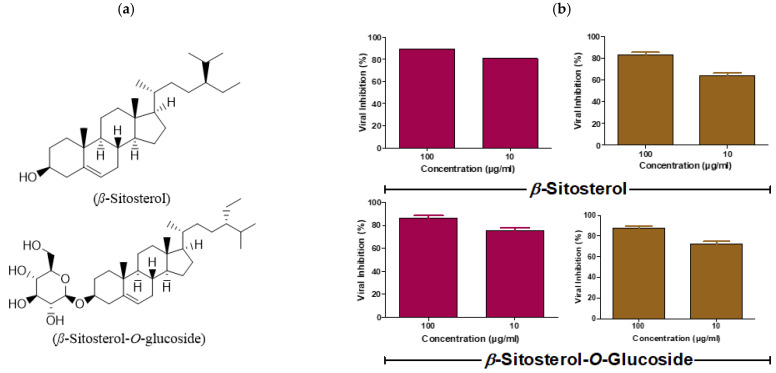
Concentration-dependent viral titer reduction for the highly promising steroids (*β*-sitosterol and *β*-sitosterol-*O*-glucoside) as determined via PRA. (**a**) Chemical structure of *β*-sitosterol and *β*-sitosterol-*O*-glucoside; (**b**) Validation of the anti-influenza efficacy for both compounds were evaluated against the A/H1N1 (dark pink) and the A/H5N1 (light brown), and GraphPad Prims 5.01 software was used to plot the viral inhibition percentages against compound concentrations.

The plaque reduction assay was simply employed to validate the anti-IAV efficacy for the highly promising steroids *β*-sitosterol and *β*-sitosterol-*O*-glucoside (Figure 3a), based on their extremely high SI values as compared to the reference drug. Strikingly, the plaque reduction percentages clearly elucidated the anti-IAV potential against both influenza subtypes (H1N1 and H5N1) for both compounds where the viral titers had been lowered using low non-cytotoxic concentrations from the tested steroids (Figure 3b). These data support the hypothesis that the investigated steroids are considered as lead compounds to target influenza disease in both human and avian hosts.

### 3.3. β-Sitosterol and β-Sitosterol-O-Glucoside affects IAV at Multiple Stages of the Virus Replication Cycle

Noticeably, any antiviral compound works against the virus through three major specific mechanisms as previously described. In this context, we aimed to explore at which step in the influenza virus replication cycle the highly effective *β*-sitosterol and *β*-sitosterol-*O*-glucoside lowered the titer of A/H1N1 virus. Curiously, both compounds exhibited their anti-influenza potency through viricidal actions where they directly target the human seasonal influenza viral particles away from the host MDCK cells (Table 3).

### 3.4. Docking studies

To understand the obtained antiviral activities at a molecular level, *β*-sitosterol and *β*-sitosterol-*O*-glucoside were docked against different viral protein targets. These proteins are influenza hemagglutinin H1 mutant DH1E (PDB ID: 5VMG, resolution: 2.45 Å), neuraminidase from influenza A/H1N1 virus (PDB ID: 3TI5, resolution: 1.90 Å), influenza proton channel M2 protein (PDB ID: 2RLF), and hemagglutinin head epitope of influenza H1N1 virus (PDB ID: 7MEM). 6′-Sialyl-***N***-acetyllactosamine, zanamivir, and rimantadine were used as reference molecules against hemagglutinin, neuraminidase, and M2 proteins, respectively. In the docking studies, it depended on both binding mode and binding energy to investigate the efficiency of binding against the active sites (Table 4).

#### 3.4.1. Validation

**Figure 4:**
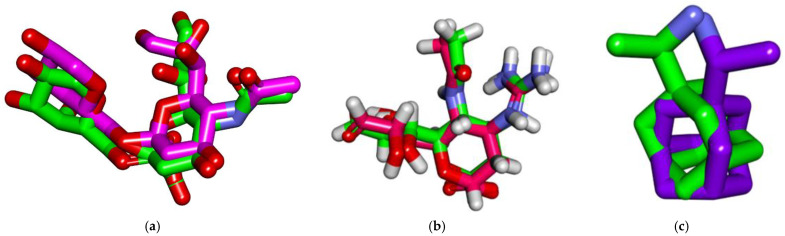
(**a**) Superimposition of the cocrystallized ligand (6’-sialyl-***N***-acetyllactosamine) of influenza hemagglutinin H1 mutant DH1E (carbon atoms in green) and the docked pose of the same ligand (carbon atoms in pink). (**b**) Superimposition of the cocrystallized ligand (zanamivir) of influenza A/H1N1 neuraminidase (carbon atoms in green) and the docked pose of the same ligand (carbon atoms in red). (**c**) Superimposition of the cocrystallized ligand (Rimantadine) of influenza proton channel M2 (carbon atoms in green) and the docked pose of the same ligand (carbon atoms in violet).

To validate the docking process, docking procedures were performed for the cocrystallized ligands in the active sites of influenza hemagglutinin H1 mutant DH1E, influenza A/H1N1 neuraminidase, and proton channel M2 utilizing MMFF94X as a force field and ASE as a scoring function allowed for the protocol’s validation. The small RMSD values between the docked poses and the cocrystallized ligands during the validation step indicated the feasibility of the used methodology for the intended docking experiments (1.14, 0.95, and 1.4 Å for influenza hemagglutinin H1 mutant DH1E, influenza A/H1N1 neuraminidase, and proton channel M2, respectively) (Figure 4).

#### 3.4.2. Docking Studies against Influenza Hemagglutinin H1 Mutant DH1E

**Figure 5:**
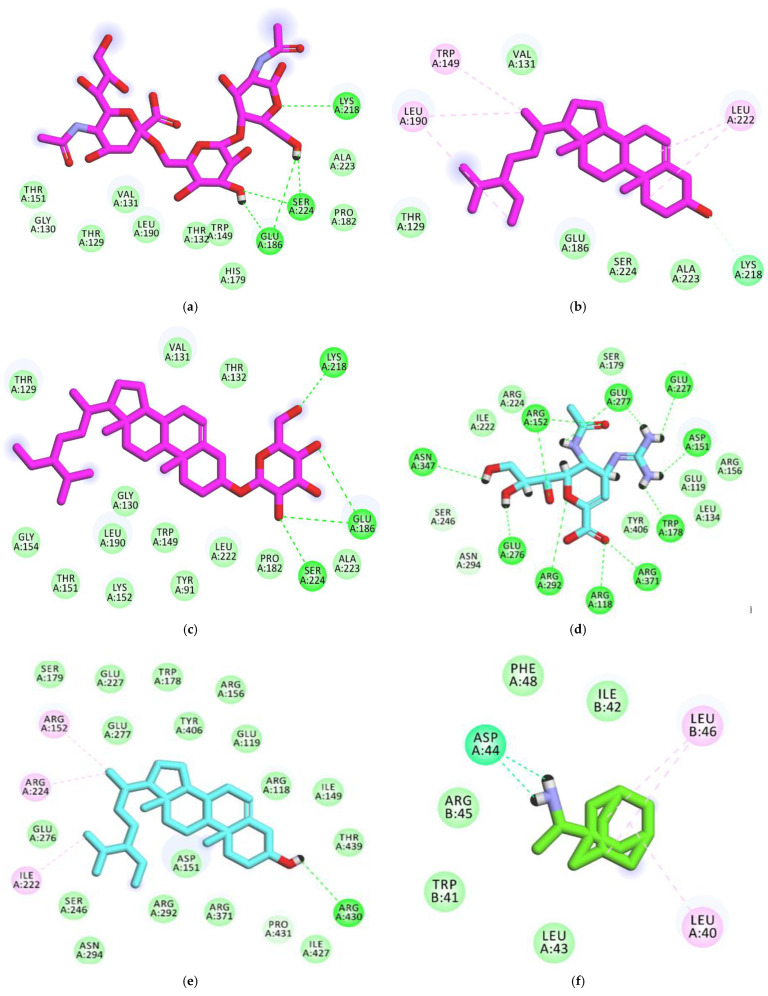

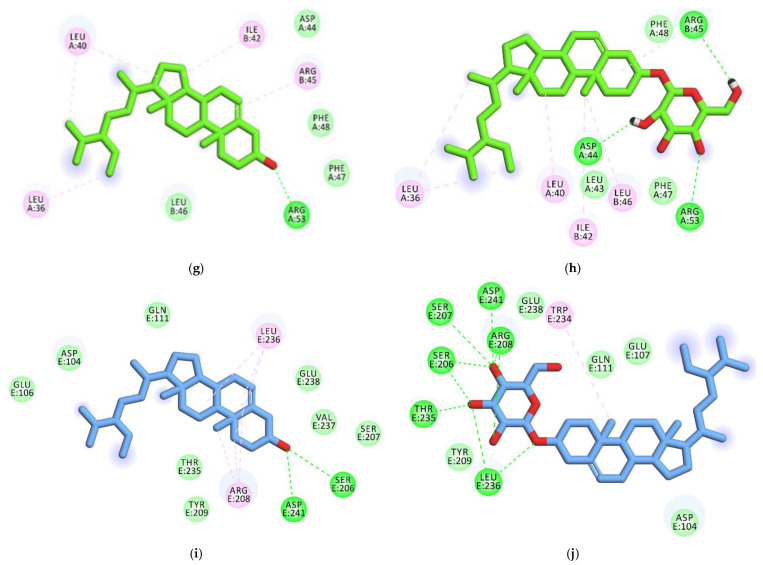
(**a**) Two-dimensional image of 6′-Sialyl-*N*-acetyllactosamine docked into the active site of influenza A/H1N1 hemagglutinin; (**b**) 2D of β-sitosterol docked into the active site of influenza A/H1N1 hemagglutinin; (**c**) 2D of β-sitosterol-O-glucoside docked into the active site of influenza A/H1N1 hemagglutinin; (**d**) 2D of the cocrystallized ligand (Zanamivir) docked into the active site of influenza A/H1N1 neuraminidase; (**e**) 2D of β-Sitosterol docked into the active site of influenza A/H1N1 neuraminidase; (**f**) 2D of the cocrystallized ligand (Rimantadine) docked into the active site of influenza proton channel M2 protein; (**g**) 2D of β-sitosterol docked into the active site of influenza proton channel M2 protein; (**h**) 2D of β-sitosterol-o-glucoside docked into the active site of influenza proton channel M2 protein; (**i**) 2D of β-sitosterol docked into hemagglutinin head epitope; (**j**) 2D of β-sitosterol-o-glucoside docked into hemagglutinin head epitope.

The reference molecule (6′-Sialyl-***N***-acetyllactosamine) showed a binding score of -5.66 kcal/mol against influenza hemagglutinin H1 mutant DH1E. The *N*-((2S,3R,4R,5S,6R)-2,4,5-trihydroxy-6-(hydroxymethyl)tetrahydro-2*H*-pyran-3-yl)acetamide moiety was oriented into the first pocket of the active site, forming three hydrogen bonds with Lys218, Glu186, and Ser224. In addition, the (2R,3R,4S,5S,6R)-6-methyltetrahydro-2H-pyran-2,3,4,5-tetraol moiety was oriented into the second pocket, forming two hydrogen bonds with Glu186 and Ser224. Furthermore, the (2S,4S,5R,6R)-5-acetamido-4-hydroxy-2-methoxy-6-((1S,2R)-1,2,3-trihydroxypropyl)tetrahydro-2*H*-pyran-2-carboxylic acid moiety occupied the third pocket, in close contact with Leu190, Val131, Thr129, Gly130, Thr151, and Glu127 (Figure 5A, Supplementary Figure S2).

Regarding the *β*-sitosterol, it exhibited a binding affinity of −6.40 Kcal/mol against influenza hemagglutinin H1 mutant DH1E. The hydroxyl group formed a hydrogen bond with Lys218. Steroid moiety formed two hydrophobic interactions with Leu222. The side chain ((*S*)-3-ethyl-2-methylheptane) formed three hydrophobic interactions with Trp149 and Leu190 (Figure 5B, Supplementary Figure S3).

Concerning *β*-sitosterol-*O*-glucoside, it showed a binding score of −6.78 Kcal/mol against influenza hemagglutinin H1 mutant DH1E. The sugar moiety formed four hydrogen bonds with Lys218, Glu186, and Ser224. The steroid moiety and the aliphatic side chain formed a close contact with Thr132, Val131, Thr129, Glu127, Gly154, Gly130, Trp149, Leu222 (Figure 5C, Supplementary Figure S4).

#### 3.4.3. Docking Studies against Influenza A/H1N1 Neuraminidase

The cocrystallized ligand (zanamivir) showed a binding sore of −19.28 kcal/mol against influenza A/H1N1 neuraminidase. The guanidine and acetamide moieties were oriented into the first pocket of the active site, forming six hydrogen bonds with Glu277, Trp178, Glu227, Arg152, and Asp151. The cyclohex-1-ene-1-carboxylic acid moiety was oriented into the second pocket, forming three hydrogen bonds with Arg371, Arg292, and Arg118. Furthermore, the propane-1,2,3-triol moiety occupied the third pocket, forming three hydrogen bonds with Glu276, Arg152, and Asn347 (Figure 5D, Supplementary Figure S5).

The results of docking studies revealed the correct binding mode of *β*-sitosterol against the active site of influenza A/H1N1 neuraminidase. In detail, *β*-sitosterol exhibited a binding affinity of −29.4003506 Kcal/mol against influenza A/H1N1 neuraminidase. The hydroxyl group formed a hydrogen bond with Arg430. The side chain ((S)-3-ethyl-2-methylheptane) formed three hydrophobic interactions with Arg152, Arg224, and Ile222 (Figure 5E, Supplementary Figure S6).

#### 3.4.4. Docking Studies against Influenza Proton Channel M2

The cocrystallized ligand (Rimantadine) showed a binding sore of -9.96808243 kcal/mol against influenza proton channel M2 protein. The ethanamine moiety was oriented into the deep pocket of the receptor, forming two hydrogen bonds with Asp44. The adamantane moiety was oriented into the outer region of the active site, forming three hydrophobic interactions with Leu46 and Leu40 (Figure 5F, Supplementary Figure S7).

In respect of *β*-sitosterol, it exhibited a binding affinity of -10.97 Kcal/mol against influenza proton channel M2 protein. The hydroxyl group formed a hydrogen bond with Arg430. The side chain ((S)-3-ethyl-2-methylheptane) formed three hydrophobic interactions with Arg152, Arg224, and Ile222 (Figure 5G, Supplementary Figure S8).

Respecting *β*-sitosterol-*O*-glucoside, it showed a binding score of −10.75 Kcal/mol against influenza M2 protein. The sugar moiety formed three hydrogen bonds with Arg45, Arg53, and Asp44. The steroid moiety and the aliphatic side chain formed six hydrophobic interactions with Arg45, Leu46, Ile42, Leu40, and Leu36 (Figure 5H, Supplementary Figure S9).

#### 3.4.5. Docking Studies against Hemagglutinin Head Epitope of Influenza A/H1N1 Virus

The docking of both *β*-sitosterol and *β*-sitosterol-*O*-glucoside against hemagglutinin head epitope of influenza A/H1N1 virus revealed that *β*-sitosterol (binding score = −10.90) has a better binding mode and binding energy than *β*-sitosterol-*O*-glucoside (binding score = −8.97).

For *β*-sitosterol, it was completely buried inside the hemagglutinin head epitope. It formed two hydrogen bonds with Ser206 and Asp241. In addition, the steroid moiety formed six hydrophobic interactions with Leu236 and Arg208 (Figure 5I, Supplementary Figure S10).

Regarding *β*-sitosterol-*O*-glucoside, it exhibited a shallow binding with hemagglutinin head epitope. Only the sugar moiety was oriented into the pocket of hemagglutinin head epitope, forming nine hydrogen bonds with Arg208, Asp241, Ser207, Ser206, Thr235, and Leu236 (Figure 5J, Supplementary Figure S11). It can be concluded that *β*-sitosterol has a good chance to bind and block the hemagglutinin head epitope of the influenza A/H1N1 virus.

### 3.5. Phytoestrogen β-Sitosterol Is Likely to Hinder IAV Replication in an Estrogen-Like Mode

**Figure 6:**
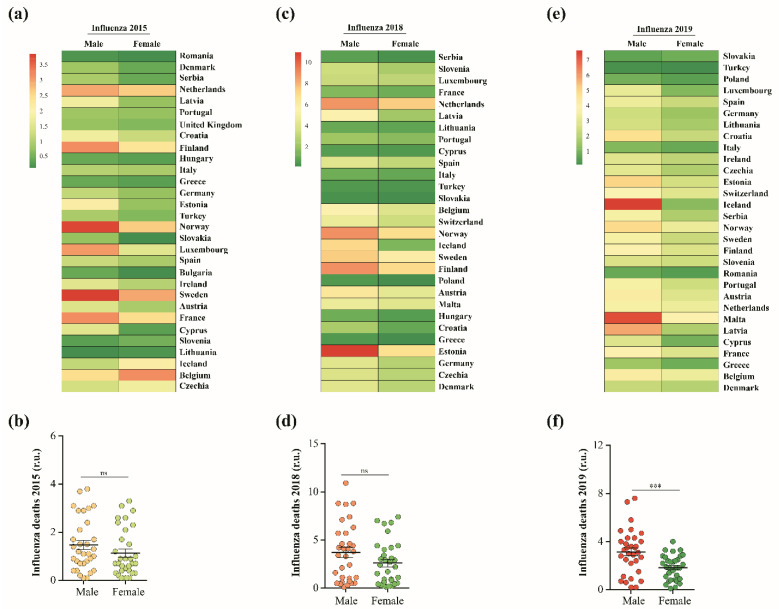
Sex-disaggregated data for influenza deaths during 2015, 2018, and 2019: (**a**) heatmap depicting sex differences in the death rates of influenza during 2015 in 30 EU countries; (**b**) mean differences between datasets representing both sexes in a; (**c**) heatmap depicting sex differences in the death rates of influenza during 2018 in 29 EU countries; (**d**) mean differences between datasets representing both sexes in c; (**e**) heatmap depicting sex differences in the death rates of influenza during 2019 in 30 EU countries; (**f**) mean differences between datasets representing both sexes in e. Results (**b**,**d**,**f**) are expressed as mean ± SEM. ns denotes no significance and *** *p* ≤ 0.001.

Recent studies discussing the existence of male-biased mortality in sex-disaggregated data of COVID-19 suggest that the female sex hormone estrogen is likely to contribute to this phenomenon [113]. Interestingly, by analyzing the sex-disaggregated datasets covering influenza deaths during 2015, 2018, and 2019 in 32 European countries with available indexed data according to the EU’s standardized death rate for pulmonary diseases (Figure 6a,c,e), it was remarkable that the standardized death rates of males by influenza illness were higher during 2015 and 2018, but not significant compared to females for the same years. The mean death rates for males during 2015 and 2018 were 1.5 ± 0.1932 (mean ± SEM), and 3.7 ± 0.537, respectively, and the influenza death rates for females were 1.1 ± 0.1755 and 2.6 ± 0.4021 during 2015 and 2018, respectively (Figure 6b,d). The male death rates of influenza during 2019 were 1.72 higher than females, with mean values of 3.1 ± 0.3304 for males and 1.8 ± 0.1813 for females, demonstrating a significant difference between sexes and substantially biased mortality towards males (Figure 6f).

**Figure 7:**
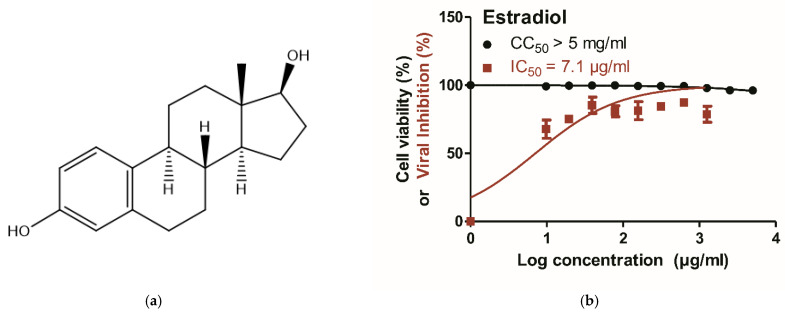
Structural similarity of *β*-sitosterol with estradiol and potential anti-influenza activity. (**a**) Chemical structure of the potent human estrogen form; namely estradiol; (**b**) the cytotoxicity of estradiol as expressed in CC_50_ and the antiviral activity against A/H1N1 as expressed as an IC_50_ value. GraphPad Prism 5.01 software was used to analyze the nonlinear regression, while the CC_50_ and IC_50_ were determined by plotting log inhibitor against normalized response (variable slope).

The phytoestrogen *β*-sitosterol and its derivative *β*-sitosterol-*O*-glucoside are structurally similar to estradiol, which is an estrogen steroid hormone and the major female sex hormone. The structural similarity between *β*-sitosterol and estradiol (Figure 3a and Figure 7a) and the observed male-biased mortality in sex-disaggregated data of influenza deaths (Figure 6a–f) recommended the testing of the anti-influenza activity of estradiol. Interestingly, estradiol has high safety at a wide range of concentrations (CC_50_ > 5 mg/mL), with robust antiviral activity against seasonal influenza A/H1N1 virus (IC_50_ = 7.1 µg/mL) (Figure 7b). Conclusively, estradiol showed antiviral activity against the influenza A/H1N1 virus in vitro but with a lower IC_50_ value, when compared with *β*-sitosterol.

## 4. Discussion

The COVID-19 pandemic has highlighted the need for novel and safe antivirals that can combat emerging and reemerging respiratory RNA viruses [114]. Traditional medicine, which is ultimately based on plants and their secondary metabolites (i.e., phytochemicals), is considered one of the main therapeutic approaches to cure different diseases such as viral diseases, cancer, and chronic diseases (e.g., diabetes and heart diseases) [115]. In the last two decades, about 34% of the total newly approved drugs were naturally derived chemical substances [116]. Accordingly, we aimed at repurposing naturally occurring chemical compounds with different chemical classes as new antiviral candidates to help in controlling the respiratory viral infections, specifically avian and human IAVs.

Herein, we successfully unmasked the high antiviral potential of a well-known phytosterol, namely *β*-sitosterol and its *O*-glycoside derivative, against seasonal human influenza and avian viruses (H1N1 and H5N1 subtypes) with high SI values as compared to the standard FDA-approved anti-influenza drug (zanamivir). Additionally, the results revealed that low concentrations of this steroid compound (10–100 µg/mL) could inactivate up to 90% of viral infectivity through direct viricidal action on the A/H1N1 subtype.

A recent in silico study suggested that *β*-sitosterol could suppress viral infection with SARS-CoV-2 through targeting the receptor-binding domain (RBD) of the spike glycoprotein [54]. Previous investigations have shown also that *β*-sitosterol exhibits antioxidant, anti-inflammatory, anticancer, and in vitro antiviral activity against white spot syndrome virus [51,52,53,54]. However, antiviral activities against IAVs, to the best of our knowledge, have not yet been reported.

In addition to *β*-sitosterol, our results also showed robust antiviral activities of *β*-sitosterol-*O*-glucoside (Sitogluside or Daucosterol) against both tested IAVs, mainly via direct viricidal action. In addition, *β*-sitosterol-*O*-glucoside could induce up to 90% viral inhibition with very low concentration (10–100 µg/mL). The reported biological activities for *β*-sitosterol-*O*-glucoside have shown that it possesses anticancer and antidiabetic activities [55,57]. An in silico study for the antiviral effects of *β*-sitosterol-*O*-glucoside against SARS-CoV-2 have shown that sitogluside possesses inhibitory effects against the tested virus, as demonstrated by virtual study that revealed the capability of this compound to target the surface RBD of spike glycoprotein on the viral particle [56]. This emphasizes the observed virucidal mode of antiviral action of the investigated phytosterols against IAVs.

On the same hand, naringin proved to exhibit antiviral potential against human immunodeficiency virus type 1 (HIV-1), and an in silico study showed its potential to work against SARS-CoV-2, as it has high binding affinity to the virus’s main protease [70,72]. Herein, naringin showed moderate antiviral activity against the influenza A/H1N1 virus, indicating its antiviral activity but to certain extent. Likewise, Gao and his colleagues have recently showed that digitonin has antiviral activity against Zika virus with low IC_50_ of 7.91 µM [117]; however, in this study, we showed that the digitonin has moderate antiviral activity against influenza A/H1N1 virus.

On the other hand, previous investigations on silybin using MTT assay to estimate the cytotoxicity and the SRB method to evaluate its antiviral assay (against A/ShanTou/169/06 (H1N1)) showed that silybin has high very low cytotoxicity on MDCK cells with a CC_50_ > 400 µM and low antiviral activity, with an EC_50_ value of 70.78±8.11 µM [28].. Our findings also proved the nontoxic effects of silybin on MDCK cells (CC_50_ value of 9.48 mg/mL) and poor anti-influenza potential against the human influenza A/H1N1 virus. However, 7-hydroxy flavanone showed antiviral activity against enterovirus type A-71 (EV-A71) [31], but it showed poor antiviral activity against A/H1N1 in this study. Likewise, previous reports proved the antiviral properties of pinocembrin against different viruses, including SARS-CoV-2, Zika virus (ZIKV), Herpesvirus type-1 (HSV-1), and Canine distemper virus [32,33,35], whereas in our findings, pinocembrin showed poor anti-H1N1 activity. Recently, a molecular docking (MD) simulation study also showed that kaempferitrin may possess antiviral potential against SARS-CoV-2 [76], whilst in this study, it showed moderate antiviral activity against the seasonal influenza A/H1N1 virus, with IC_50_ value of 47.8 µg/mL. In a study conducted by Won-Kyung Cho and his colleagues [81], they reported that isoquercitrin exert antiviral activity against different influenza A strains, namely (A/PR8/34(H1N1))-GFP, A/PR8/34 viruses, and HBPV-VR-32 (H3N2); however, information regarding compound cytotoxicity and inhibitory concentration values were not provided. We found that isoquercitrin is relatively safe on MDCK cells with CC_50_ value of 0.71 mg/mL as compared to the reference drug with poor antiviral potential against the seasonal influenza (H1N1) virus (IC_50_ value of 167 µg/mL). Nevertheless, the wide disparity in IC_50_ values of some tested phytochemicals could be due to a number of factors such as extraction technique, cellular and viral model variations, or antiviral methods used.

Several studies reported the viricidal potentialities of different molecules through the inhibition of the hemagglutinin protein [118,119,120]. Accordingly, we investigated the abilities of *β*-sitosterol and *β*-sitosterol-*O*-glucoside against such protein. Both compounds exhibited perfect binding combined with excellent energy comparing the reference compound. These findings suggest that *β*-sitosterol and *β*-sitosterol-*O*-glucoside exert their strong viricidal activities through direct inactivation of the surface hemagglutinin protein, hindering receptor binding and the subsequent viral entry process [7]. The viral neuraminidase (NA) is present on the surface of influenza viruses, allowing the virus to escape from the host cell [121]. Additionally, the interesting transmembrane protein (M2) of the influenza virus functions as a tiny proton channel in the viral envelope that has an essential role following virus endocytosis [7,122]. To understand the inhibitory effects against viral replication, molecular docking studies have been performed for both compounds against NA and M2 proteins and revealed their perfect binding modes as well as their low binding energies. These data confirmed that *β*-sitosterol and *β*-sitosterol-*O*-glucoside can affect IAV at different stages during the influenza virus replication cycle.

During the COVID-19 pandemic, several studies have discussed the male-biased mortality in sex-disaggregated data of COVID-19, suggesting that the female sex hormone estrogen is likely to contribute to partially protecting and alleviating disease severity or progression [113]. Similarly, by analyzing the distribution of the differential significance between numbers of influenza cases and deaths within three influenza seasons in Europe, we also found male-biased mortality in sex-disaggregated data of influenza viruses with significant differences towards males during the 2019 influenza season. Interestingly, phytoestrogens, including *β*-sitosterol, are structurally similar to endogenous estrogens such as estradiol [123]. This structural similarity may explain the ability of both estrogens to control IAV replication via similar mechanisms. It is worth mentioning that the phytoestrogen *β*-sitosterol has more potent antiviral activity when compared with the active form of the female endogenous estrogen, but with fewer expected side effects and high biological side benefits [124,125].

## 5. Conclusions

Conclusively, our study provides a proof-of-principle demonstration that *β*-sitosterol (Phytosterol) and *β*-sitosterol-*O*-glucoside (Sitogluside) significantly have high in vitro antiviral potential against avian and human IAVs. Molecular docking studies were performed and suggested that *β*-sitosterol and *β*-sitosterol-*O*-glucoside exerted viricidal activities by binding to hemagglutinin protein. The *β*-sitosterol could also inhibit viral replication via interfering with viral neuraminidase and M2 proteins of IAV. This study also highlighted the possible estrogen-like effect of *β*-sitosterol due to the structural similarities between the two molecules and proved the anti-influenza activity of estradiol as the most active form of estrogen.

## Figures and Tables

**Table 1:** The chemical classification and biological activities of the phytochemicals and drug control used in this study.

Compound	CAS No.	Class	Reported Biological Activities	Reference
Silybin	22888-70-6	Flavonoids	Anti-inflammatory and antiviral	[27,28]
7-Hydroxy flavone	6665-86-7	Flavonoids	Anti-inflammatory and antiviral	[29,30,31]
Pinocembrin	480-39-7	Flavonoids	Anti-inflammatory, antiallergic, antioxidant, anticarcinogenic, and antiviral	[32,33,34,35]
Flavanone	487-26-3	Flavonoids	Anti-inflammatory	[36]
Saponin	8047-15-2	Triterpene	Antimicrobial, anticancer, antioxidant, antitumor, and antiviral	[37,38,39,40]
Lupeol	545-47-1	Triterpene	Antioxidant and anti-inflammatory, Antiviral (Lupeol synthetic derivatives)	[41,42,43]
Glucuronic acid	528-16-5	Sugar acids	Antioxidant, hepatoprotective, and antiviral	[44,45]
Galacturonic acid	9046-38-2	Sugar acids	Antiviral (As a saponin component)	[45,46,47,48]
D-sorbitol	50-70-4	Sugar alcohol Carbohydrates	Antiviral and laxative.	[49,50]
*β*-sitosterol	83-46-5	Steroids	Antioxidant, anticarcinogenic, anti-inflammatory, and antiviral	[51,52,53,54]
*β*-sitosterol-*O*-glucoside	474-58-8	Steroids	Antidiabetic, anticancer and antiviral	[55,56,57]
Ouabain	630-60-4	Steroid cardiac glycosides	Anticancer and antiviral	[58,59,60,61]
Digitonin	11024-24-1	Steroid saponin glycosides	Lipid solubilizing and antiviral	[62,63,64]
Arbutin	497-76-7	Phenolic glycosides	Antimelanogenesis, antidiuretic, and antiviral	[65,66]
D- (-) salicin	138-52-3	Phenolic glycosides	Antiviral and anti-inflammatory.	[67,68,69]
Naringin	10236-47-2	Flavonoid glycosides	Anti-inflammatory, anticancer, and antiviral	[70,71,72,73]
Kaempferitrin	482-38-2	Flavonoid glycosides	Hypoglycemic, anti-inflammatory, and antiviral	[74,75,76,77]
Isoquercitrin	482-35-9	Flavonoid glycosides	Antioxidant, antipruritic, neuroprotective, antibacterial, hepatoprotective, anti-inflammatory, and antiviral	[78,79,80,81,82]
Chrysophanic acid	481-74-3	Anthraquinones	Antiviral	[83,84]
Aloe emodin	481-72-1	Anthraquinones	Antiviral, anticancer, anti-inflammatory, and antibacterial.	[85,86,87,88,89,90,91]
O-Coumaric acid	614-60-8	Phenols	Antiadipogenesis, antioxidant, and antiviral (as a component of a plant, indirectly)	[92,93,94,95]
Vanillin	121-33-5	Phenols	Antiviral, antimicrobial, anti-inflammatory, antiapoptotic, neuroprotective, and antioxidant	[96,97,98,99,100]
Zanamivir	139110-80-8	NAIs	Anti-influenza	[101]

NAIs: neuraminidase inhibitors; CAS No.: Chemical Abstracts Service Registry Number.

**Table 2:** Selectivity indices for the screened compounds against influenza A/H1N1 and A/H5N1 subtypes.

Compound	Virus	CC_50_ (mg/mL)	IC_50_ (mg/mL)	SI
Silybin	H1N1	9.48	N/A	ND
	H5N1		N/A	ND
7-Hydroxy flavone	H1N1	5.83	0.360	16.194
	H5N1		N/A	ND
Naringin	H1N1	>10	0.0206	>485.43
	H5N1		N/A	ND
Pinocembrin	H1N1	>10	>10	>1
	H5N1		N/A	ND
Kaempferitrin	H1N1	>10	0.0478	>209.20
	H5N1		N/A	ND
Flavanone	H1N1	0.45	N/A	ND
	H5N1		N/A	ND
Isoquercitrin	H1N1	0.71	0.167	4.25
	H5N1		N/A	ND
Saponin	H1N1	>10	0.326	>30.674
	H5N1		N/A	ND
Lupeol	H1N1	0.56	0.0936	5.98
	H5N1		N/A	ND
D-Glucuronic acid	H1N1	10.26	N/A	ND
	H5N1		N/A	ND
D-Galacturonic acid	H1N1	9.61	N/A	ND
	H5N1		N/A	ND
*β*-sitosterol	H1N1	9.9	0.000975	10,154
	H5N1		0.000295	33,559
*β*-sitosterol-*O*-glucoside	H1N1	1.75	0.000719	2434
	H5N1		0.000613	2855
Ouabain	H1N1	0.176	N/A	ND
	H5N1		N/A	ND
Digitonin	H1N1	0.442	0.0215	20.56
	H5N1		N/A	ND
Chrysophanic acid	H1N1	0.0461	N/A	ND
	H5N1		N/A	ND
Aloe emodin	H1N1	2.28	0.729	3.127
	H5N1		N/A	ND
Arbutin	H1N1	>10	0.764	>13.09
	H5N1		N/A	ND
O-Coumaric acid	H1N1	9.172	NA	ND
	H5N1		N/A	ND
Vanillin	H1N1	0.242	N/A	ND
	H5N1		N/A	ND
D-sorbitol	H1N1	>10	3.11	>3.215
	H5N1		N/A	ND
D-(-) salicin	H1N1	>10	N/A	ND
	H5N1		N/A	ND
Zanamivir	H1N1	>10	0.000792	>12,626
	H5N1		0.000265	>37,736

CC_50_: half-maximal cytotoxic concentration; IC_50_: half-maximal inhibitory concentration; SI: selectivity index = CC_50_/IC_50._

**Table 3:** The mechanism of action(s) for the highly effective steroids as depicted through plaque reduction % in a concentration-dependent manner.

Steroid	Concentration (µg/mL)	Stage of Antiviral Action		
		Viral Replication	Viricidal	Viral Adsorption
*β*-sitosterol	1	31.8%	84%	28%
	10	41.2%	98.5%	37.5%
	100	52.9%	99%	52.5%
*β*-sitosterol-*O*-glucoside	1	28.7%	83.6%	35.8%
	10	37.7%	93.3%	48.3%
	100	52.8%	97.1%	56.9%

**Table 4:** Binding free energies (∆G in Kcal/mol) of *β*-sitosterol, and *β*-sitosterol-*O*-glucoside, against hemagglutinin, neuraminidase, M2, and hemagglutinin head epitope proteins as compared to the reference molecules.

Compound	Hemagglutinin	Neuraminidase	M2	Hemagglutinin Head Epitope
*β*-sitosterol	−6.40	−29.40	−10.97	−10.90
*β*-sitosterol-*O*-glucoside	−6.78	−30.13	−10.75	−8.97
6′-Sialyl-*N*-acetyllactosamine	−5.66	-	-	-
Zanamivir	-	−19.28	-	-
Rimantadine	-	-	−9.968	-

## Data Availability

Not applicable.

## Supplementary Materials

The following supporting information can be downloaded at https://www.mdpi.com/article/10.3390/vaccines11020228/s1: Figures S1–S11.
