# Supplementary material for: Phytoestrogen β-Sitosterol Exhibits Potent In Vitro Antiviral Activity against Influenza A Viruses
Source: Vaccines (Basel). 2023 Jan 19;11(2):228. doi: 10.3390/vaccines11020228 (PMC9964242; doi:10.3390/vaccines11020228)
Supplement: Supplementary file 1 [file vaccines-11-00228-s001.zip › vaccines-2139043-supplementary.pdf]

Supplementary Material

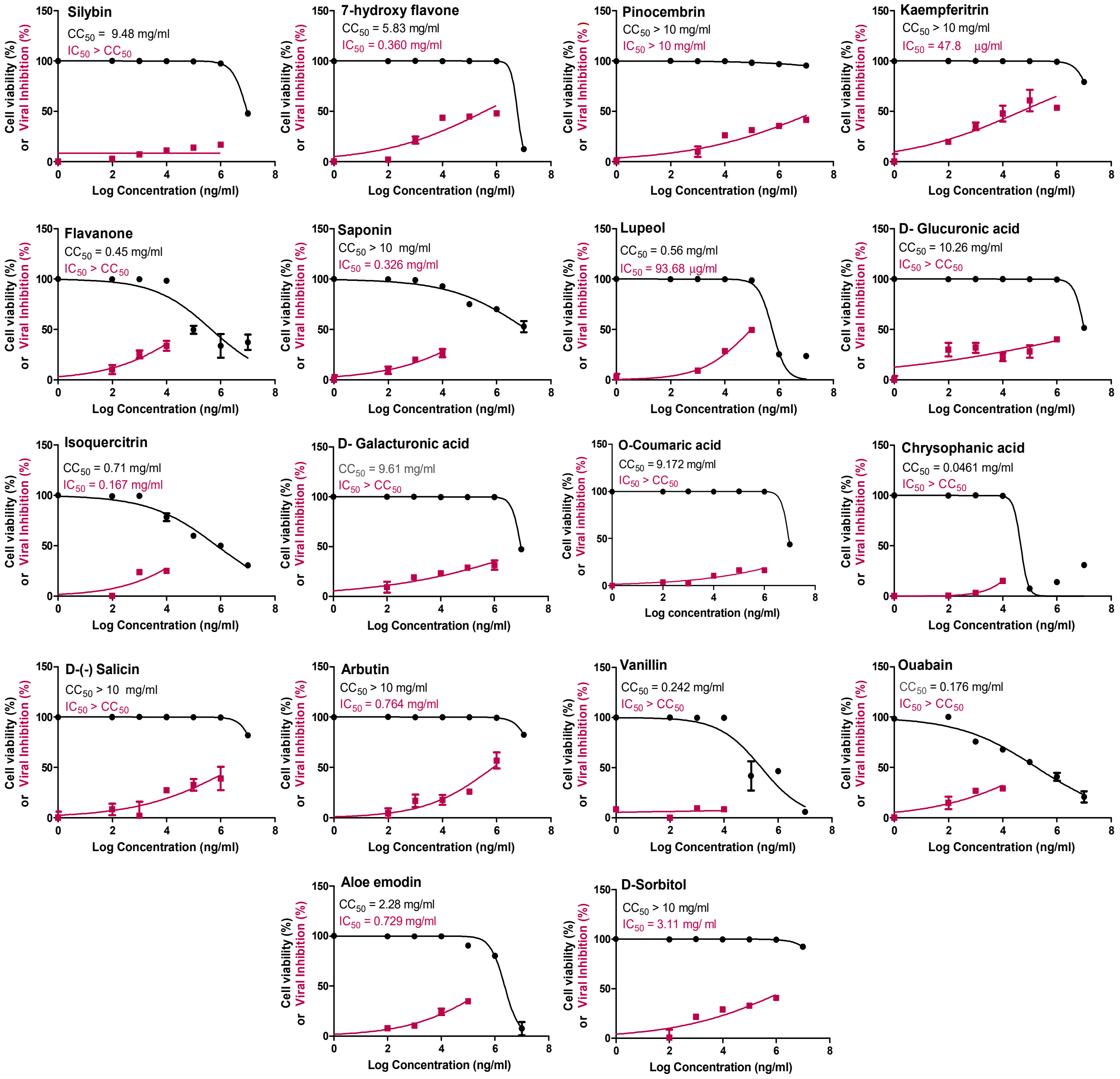

Supplementary Figure S1. The cytotoxicity as expressed in CC$_{50}$ (half maximal cytotoxic concentration) and the antiviral efficacy against A/H1N1 as expressed in IC$_{50}$ (half maximal inhibitory concentration) for the studied phytochemicals. GraphPad Prism 5.01 software was used to analyze the nonlinear regression while the CC$_{50}$ and IC$_{50}$ were determined by plotting log inhibitors against normalized response (variable slope).

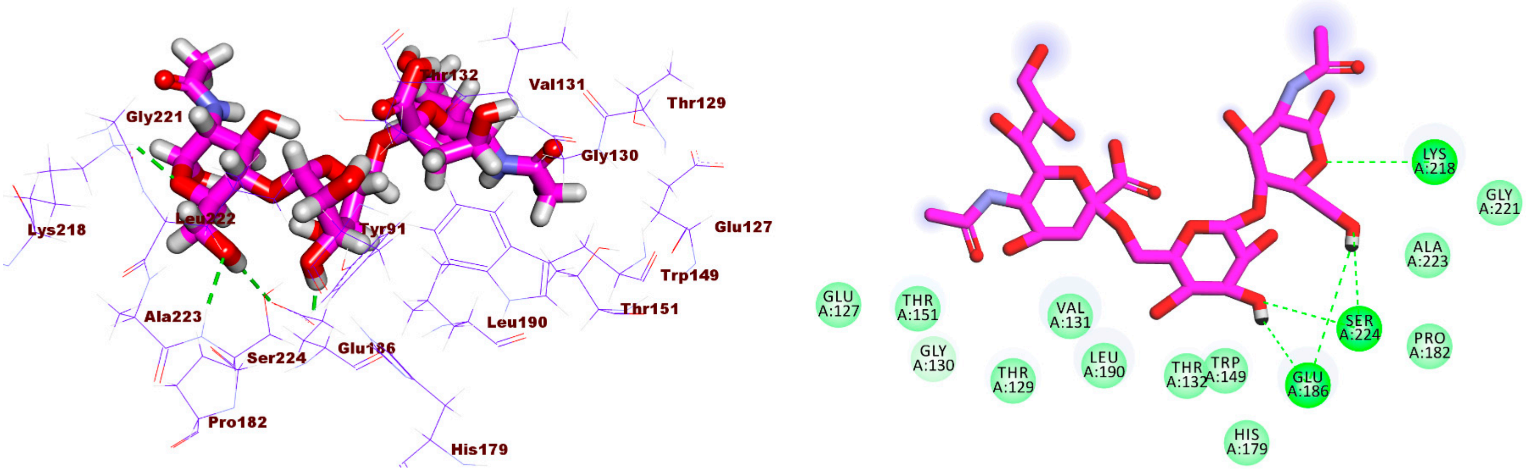

(a) (b)

Supplementary Figure S2. (**a**) 3D of 6'-sialyl-*N*-acetyllactosamine docked into the active site of influenza hemagglutinin H1 mutant DH1E. (**b**) 2D of 6'-sialyl-*N*-acetyllactosamine docked into the active site of influenza hemagglutinin H1 mutant DH1E

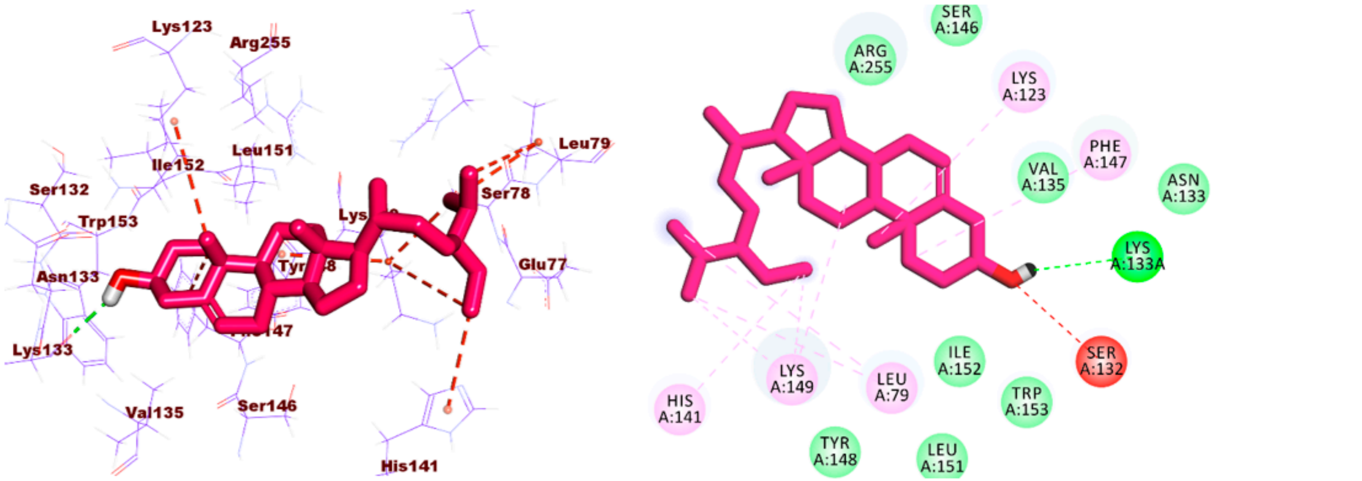

(a) (b)

Supplementary Figure S3. (**a**) 3D of *β*-sitosterol docked into the active site of influenza hemagglutinin H1 mutant DH1E (**b)** 2D of *β*-sitosterol docked into the active site of influenza hemagglutinin H1 mutant DH1E

(a)

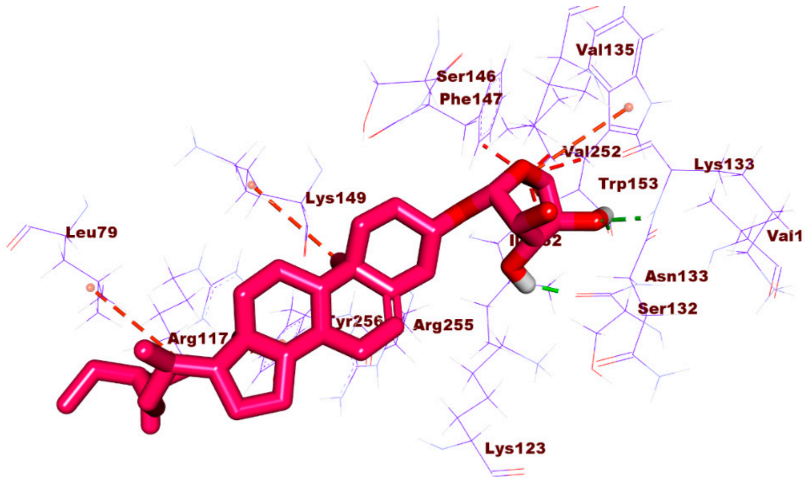

(b)

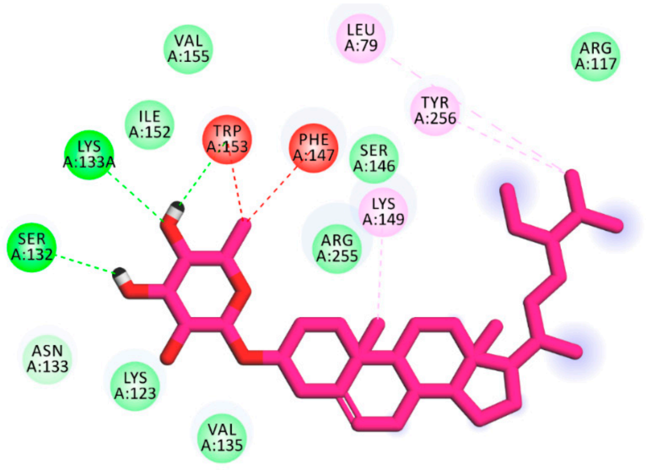

Supplementary Figure S4. (a) 3D of *β*-sitosterol-*O*-glucoside docked into the active site of influenza hemagglutinin H1 mutant DH1E. **(b)** 2D of *β*-sitosterol-*O*-glucoside docked into the active site of influenza hemagglutinin H1 mutant DH1E.

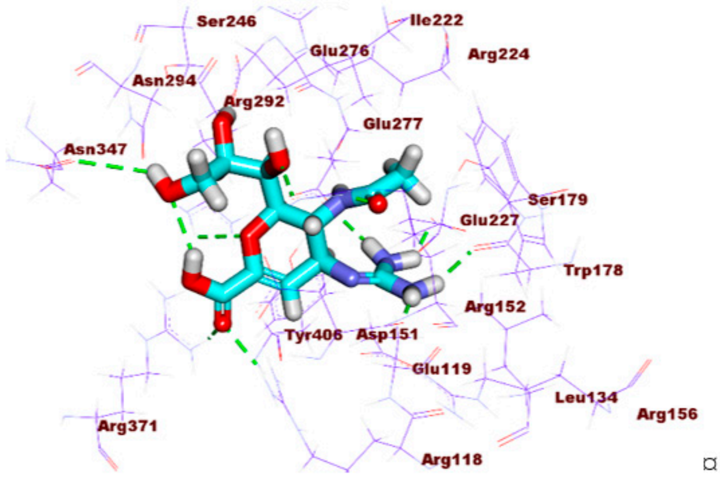

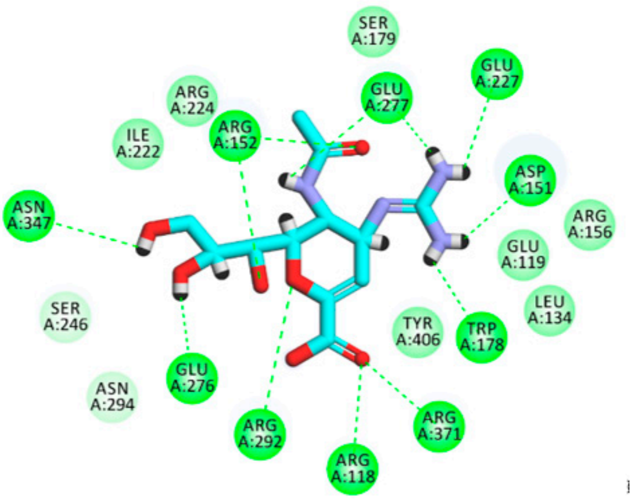

(a) **(b)**

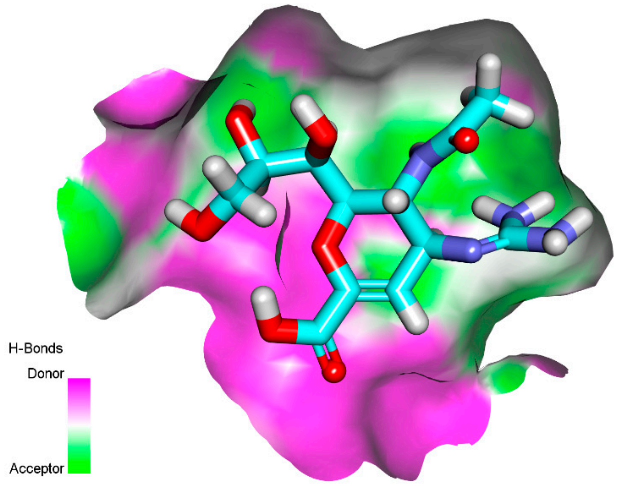

(c)

Supplementary Figure S5. (a) 3D of the co-crystallized ligand (Zanamivir) docked into the active site of influenza A/H1N1 neuraminidase (**b)** 2D of the co-crystallized ligand (Zanamivir) docked into the active site of influenza A/H1N1 neuraminidase. (**c**) Surface map of Zanamivir docked into the active site of influenza A/H1N1 neuraminidase.

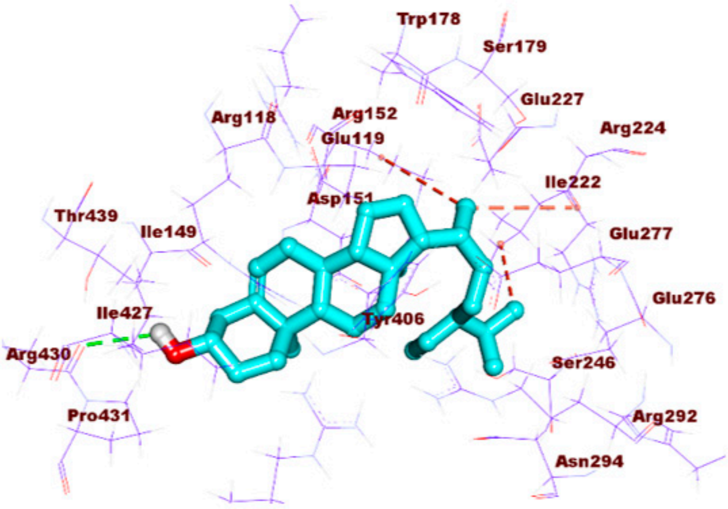

(a)

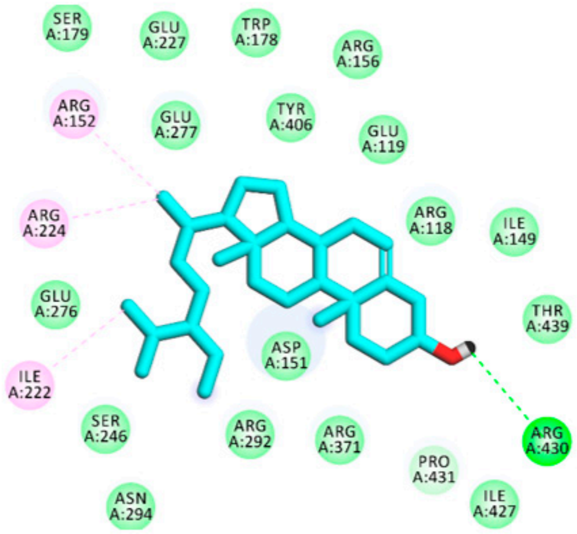

(b)

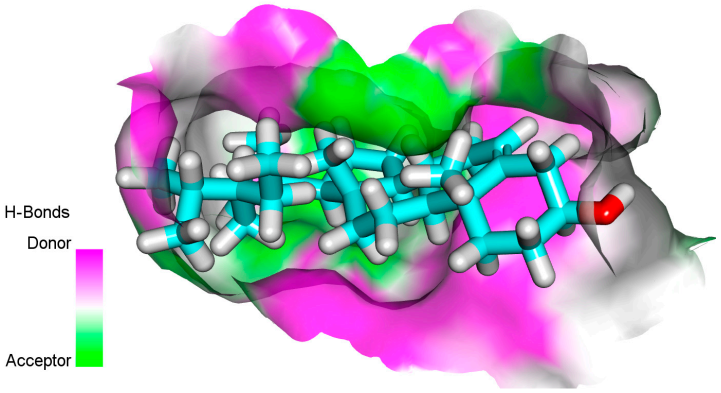

(c)

Supplementary Figure S6. (a) 3D of *β*-sitosterol docked into the active site of influenza A/H1N1 neuraminidase. **(b)** 2D of *β*-sitosterol docked into the active site of influenza A/H1N1 neuraminidase. **(c)** Surface map of *β*-sitosterol docked into the active site of influenza A/H1N1 neuraminidase.

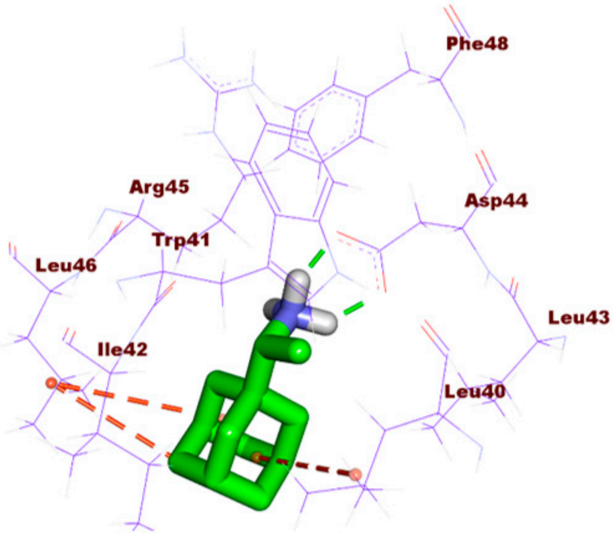

(a)

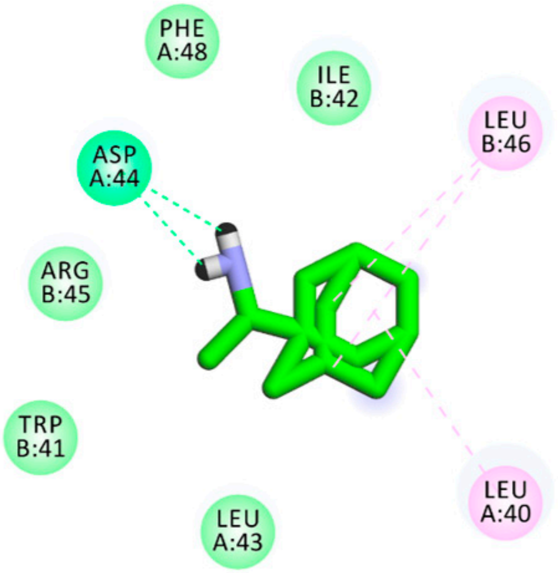

(b)

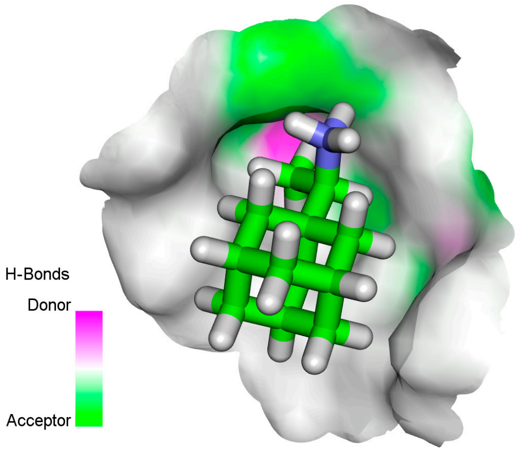

(c)

Supplementary Figure S7. (a) 3D of the co-crystallized ligand (Rimantadine) docked into the active site of influenza proton channel M2 protein. **(b)** 2D of the co-crystallized ligand (Rimantadine) docked into the active site of influenza proton channel M2 protein. **(c)** Surface map of Rimantadine docked into the active site of influenza proton channel M2 protein.

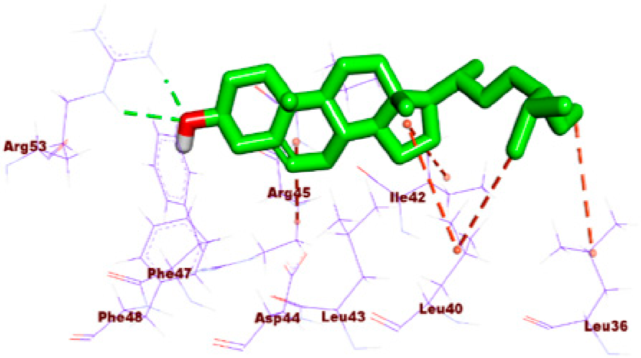

(a)

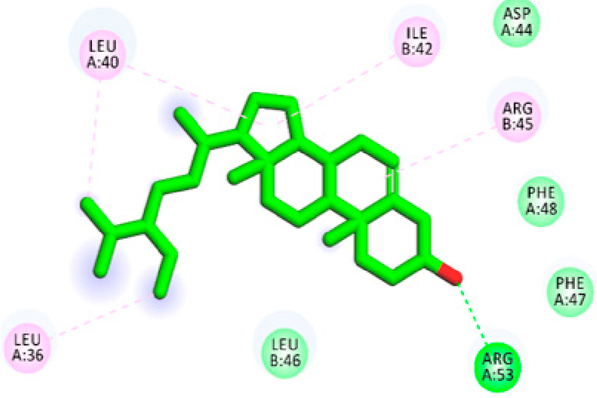

(b)

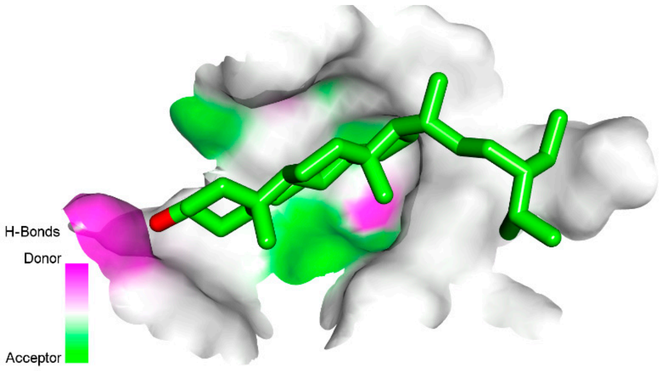

(c)

Supplementary Figure S8. (a) 3D of *β*-sitosterol docked into the active site of influenza proton channel M2 protein. **(b)** 2D of *β*-sitosterol docked into the active site of influenza proton channel M2 protein. **(c)** Surface map of *β*-sitosterol docked into the active site of influenza proton channel M2 protein.

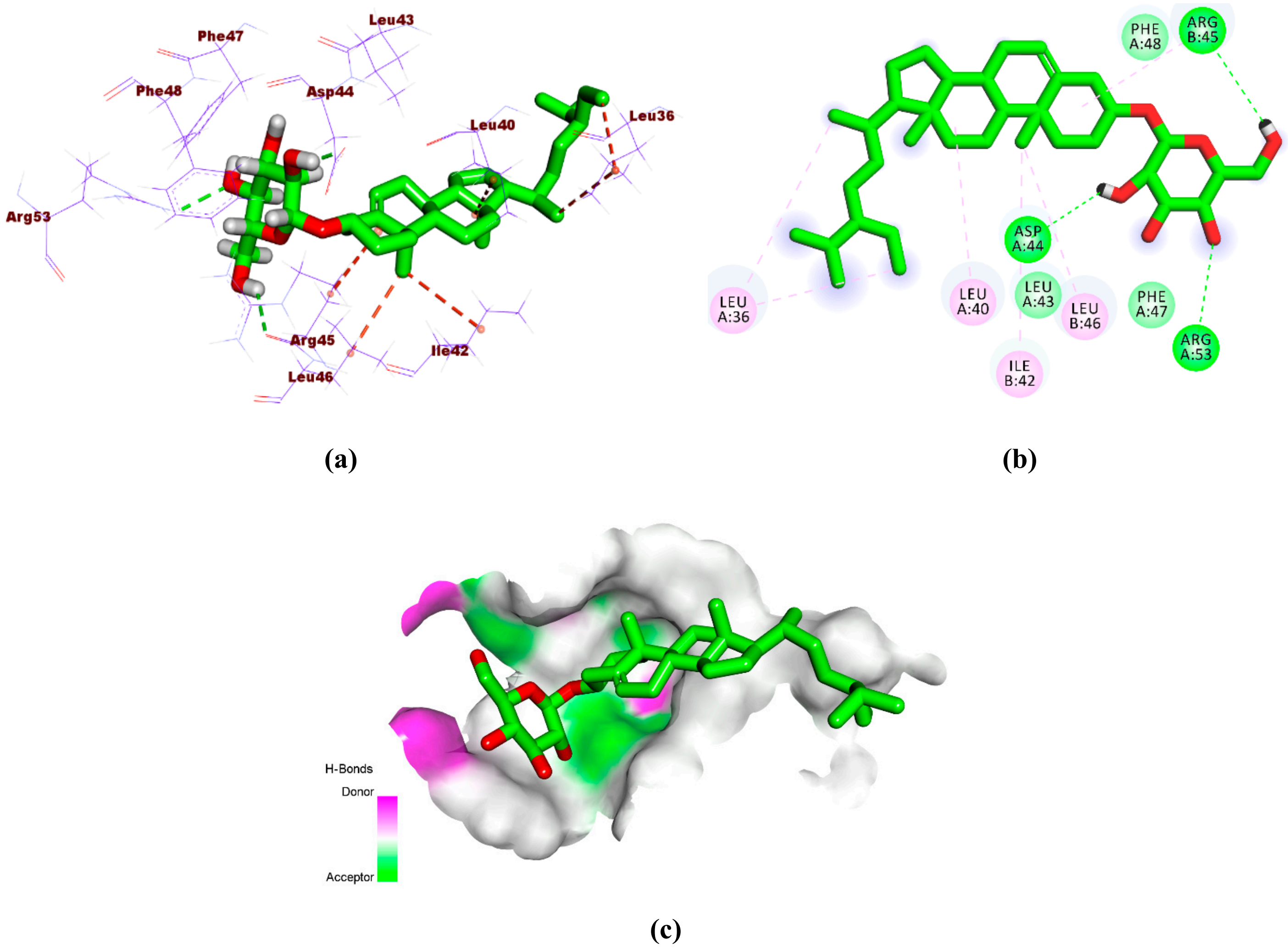

Supplementary Figure S9. (a) 3D of *β*-sitosterol-o-glucoside docked into the active site of influenza proton channel M2 protein. **(b)** 2D of *β*-sitosterol-*O*-glucoside docked into the active site of influenza proton channel M2 protein. **(c)** Surface map of *β*-sitosterol-o-glucoside docked into the active site of influenza proton channel M2 protein.

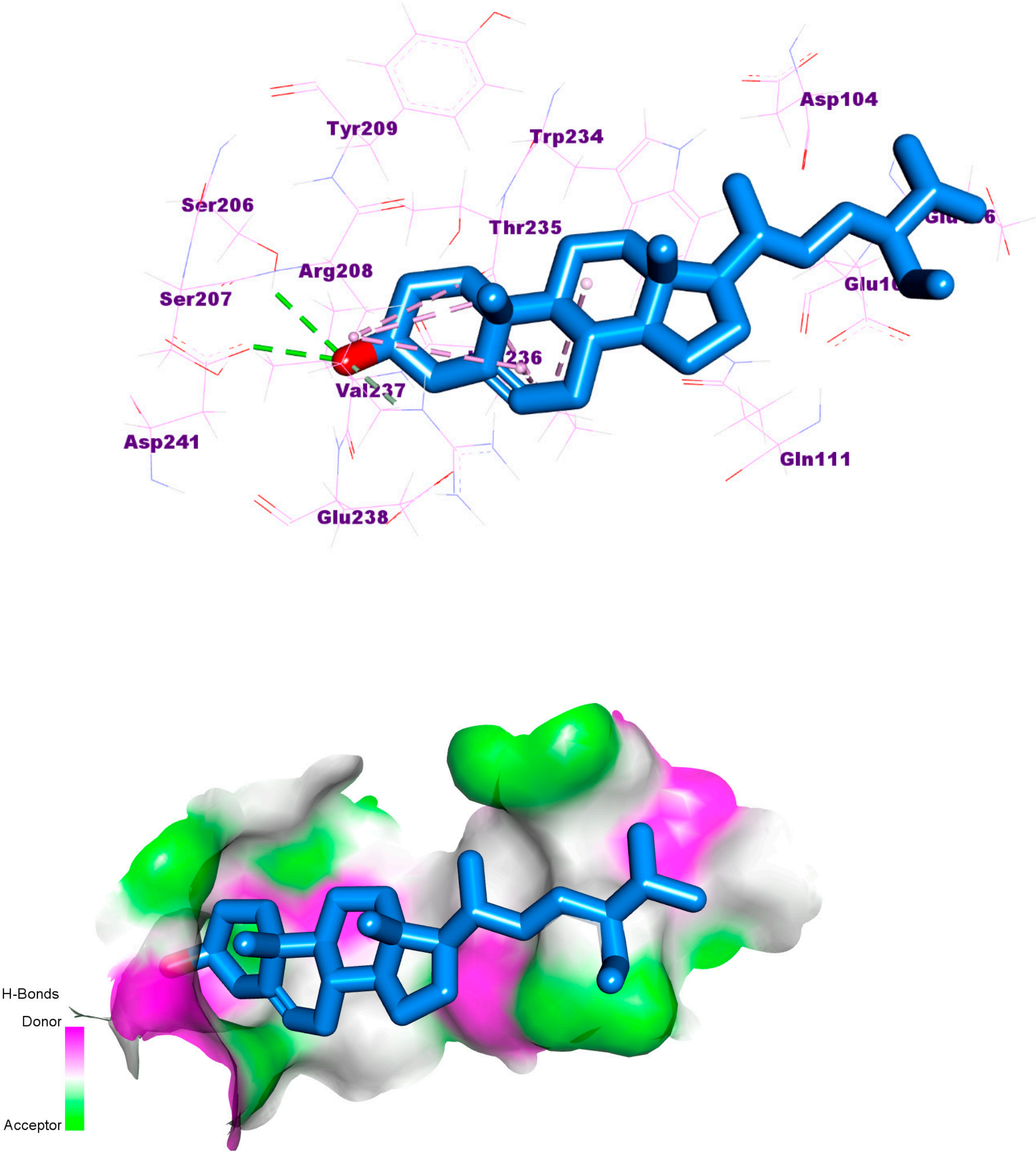

Supplementary Figure S10. 3D of *β*-sitosterol docked into hemagglutinin head epitope

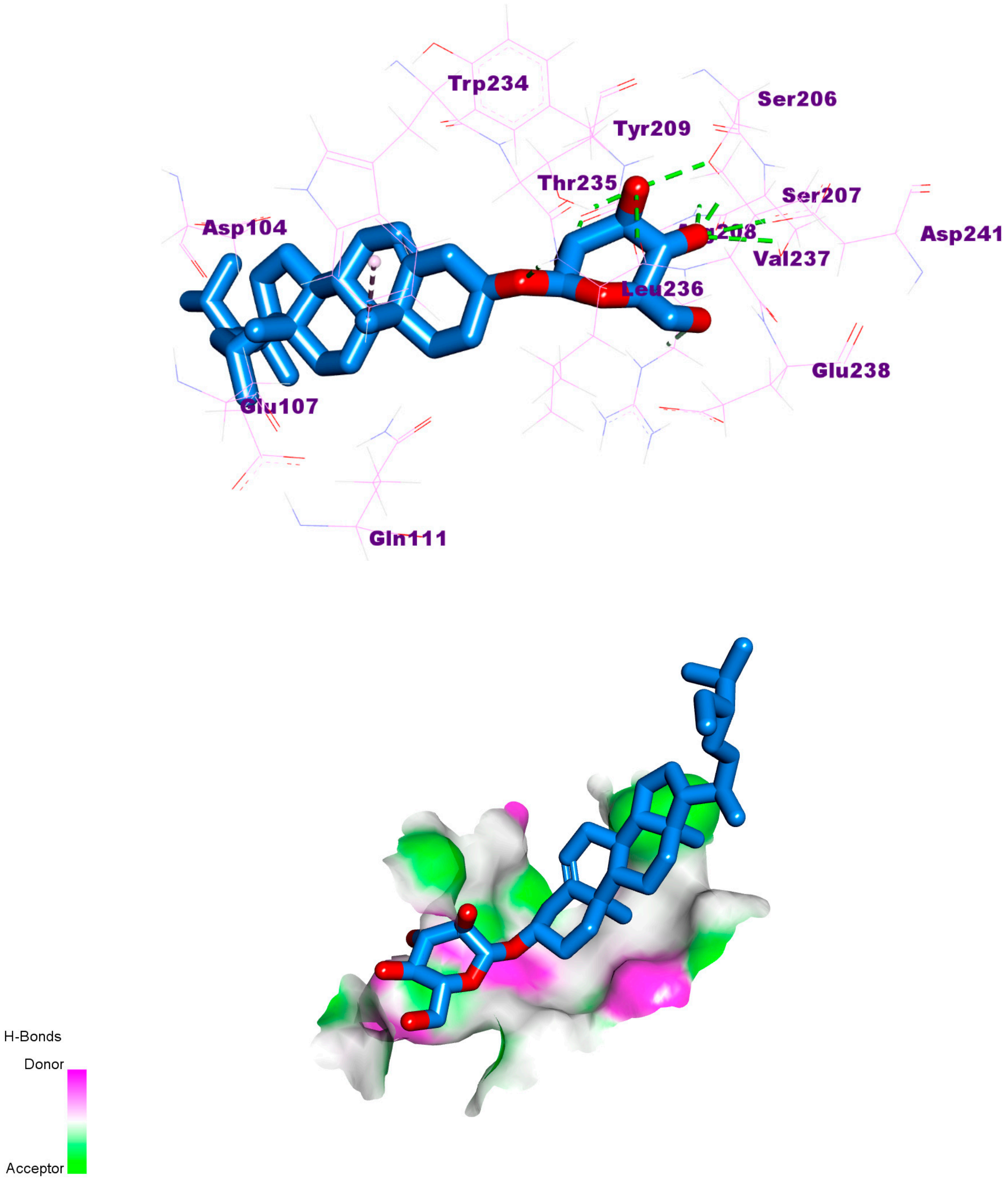

Supplementary Figure S11: 3D of *β*-sitosterol-*O*-glucoside docked into hemagglutinin head epitope.
